# Six cases of *ENPP1* pathogenic variants causing autosomal recessive hypophosphatemic rickets type 2 and generalized arterial calcification of infancy

**DOI:** 10.1093/jbmrpl/ziae174

**Published:** 2025-12-06

**Authors:** Lucy Collins, Jessica Sandy, Stephanie Ly, Kate E Lomax, Sarah Black, Fiona McKenzie, Eadaoin Hayes, Cathryn Poulton, Craig Jefferies, Wendy Hunter, Peter Simm, Christine Rodda, Andrew Biggin, Craig Munns, Aris Siafarikas

**Affiliations:** Department of Endocrinology and Diabetes, Royal Children’s Hospital%, 50 Flemington Road, Parkville, Melbourne, VIC 3052, Australia; Department of Medicine, School of Clinical Sciences, Monash University%, 246 Clayton Road, Clayton, Melbourne, VIC 3168, Australia; Institute of Endocrinology and Diabetes, The Children’s Hospital at Westmead%, Hospital Road, Westmead, Sydney, NSW 2145, Australia; Faculty of Medicine and Health, University of Sydney%, Science Road, Camperdown, Sydney, NSW 2050, Australia; Institute of Endocrinology and Diabetes, The Children’s Hospital at Westmead%, Hospital Road, Westmead, Sydney, NSW 2145, Australia; Department of Endocrinology and Diabetes, Perth Children’s Hospital%, 15 Hospital Avenue, Nedlands, Perth, WA 6009, Australia; Telethon Kids Institute, The University of Western Australia%, 15 Hospital Avenue, Nedlands, Perth, WA 6009, Australia; Department of Endocrinology and Diabetes, Perth Children’s Hospital%, 15 Hospital Avenue, Nedlands, Perth, WA 6009, Australia; Genetic Health Western Australia, King Edward Memorial Hospital%, 374 Bagot Road, Subiaco, Perth, WA 6008, Australia; Medical School, Paediatrics, University of Western Australia%, 35 Stirling Highway, Crawley, Perth, WA 6009, Australia; Department of Endocrinology and Diabetes, Perth Children’s Hospital%, 15 Hospital Avenue, Nedlands, Perth, WA 6009, Australia; Genetic Health Western Australia, King Edward Memorial Hospital%, 374 Bagot Road, Subiaco, Perth, WA 6008, Australia; Te Whatu Ora, Starship Children’s Health%, 2 Park Road, Grafton, Auckland, 1023, New Zealand; Department of Paediatrics and Liggins Institute, University of Auckland%, 34 Princes Street, Auckland Central, Auckland, 1010, New Zealand; Te Whatu Ora, Nelson Marlborough%, Waimea Road, Nelson South, Nelson, 7010, New Zealand; Department of Endocrinology and Diabetes, Royal Children’s Hospital%, 50 Flemington Road, Parkville, Melbourne, VIC 3052, Australia; Department of Paediatrics, University of Melbourne%, Parkville, Melbourne, VIC 3052, Australia; Centre for Hormone Research, Murdoch Children’s Research Institute%, 50 Flemington Road, Melbourne, VIC 3052, Australia; Department of Paediatrics, University of Melbourne%, Parkville, Melbourne, VIC 3052, Australia; Australian Institute for Musculoskeletal Research%, Level 3-4 WCHRE, 176 Furlong Road, St Albans, Melbourne, VIC 3021, Australia; Institute of Endocrinology and Diabetes, The Children’s Hospital at Westmead%, Hospital Road, Westmead, Sydney, NSW 2145, Australia; Faculty of Medicine and Health, University of Sydney%, Science Road, Camperdown, Sydney, NSW 2050, Australia; Child Health Research Centre, Faculty of Medicine, The University of Queensland%, 20 Weightman Street, Herston, Brisbane, QLD 4006, Australia; Department of Endocrinology and Diabetes, Queensland Children’s Hospital%, 501 Stanley Street, South Brisbane, Brisbane, QLD 4101, Australia; Department of Endocrinology and Diabetes, Perth Children’s Hospital%, 15 Hospital Avenue, Nedlands, Perth, WA 6009, Australia; Telethon Kids Institute, The University of Western Australia%, 15 Hospital Avenue, Nedlands, Perth, WA 6009, Australia; Medical School, Paediatrics, University of Western Australia%, 35 Stirling Highway, Crawley, Perth, WA 6009, Australia; Institute for Health Research, Notre Dame University%, 23 High Street, Freemantle, Perth, 6160WA, Australia

**Keywords:** hypophosphatemic rickets, autosomal recessive hypophosphatemic rickets type 2 (ARHR2), generalized arterial calcification of infancy (GACI), fibroblast growth factor 23 (FGF23), ectonucleotide pyrophosphate/phosphodiesterase 1 (ENPP1)

## Abstract

Autosomal recessive hypophosphatemic rickets type 2 (ARHR2) and generalized arterial calcification of infancy (GACI) occur secondary to biallelic *ectonucleotide pyrophosphate/phosphodiesterase 1* (*ENPP1*) loss-of-function pathogenic variants. GACI is a life-threatening condition, often presenting in the neonatal period with heart failure and hypertension, caused by calcification of the media in large- and medium-sized arteries. ARHR2 typically manifests later in life. Children with ARHR2 commonly exhibit short stature, rachitic skeletal changes, progressive deformities of the lower limbs, skeletal fragility and bone/muscle pain. We present six cases of homozygous pathogenic variants in the *ENPP1* gene causing ARHR2 and/or GACI.

Case 1: Presented with lower limb deformities and pain with radiological evidence of rickets. Subsequent investigations displayed aortic and pulmonary arterial calcification.

Case 2: Presented with lower limb deformities and knee pain. Confirmatory testing was undertaken following her brother’s (Case 1) diagnosis.

Case 3: The diagnosis was made antenatally. Bisphosphonate treatment was instituted in both the pre- and post-natal periods due to the presence of extensive arterial calcifications. Rickets were noted by two years of age.

Case 4: Presented with lower limb deformities and pain. There is neither any current evidence of arterial calcification nor hypertension.

Case 5: Presented at 3 mo of age in cardiogenic shock with widespread calcification of large and medium-sized arteries. Bisphosphonate treatment was instituted.

Case 6: Presented at 2 wk of age with right shoulder discomfort, with evidence of glenohumeral joint calcification. Further imaging revealed aortic, mediastinal, sternoclavicular joint and vertebral spinous process calcification.

Case 1 and 2 were also found to have a heterozygous pathogenic *ALPL* variant consistent with hypophosphatasia.

Clinical features, biochemistry, imaging and genetic analyses assist in the diagnosis of ARHR2 and GACI. Conventional therapy, oral phosphate and calcitriol for ARHR2 and bisphosphonates for GACI, have been utilized for many years. ENPP1 replacement treatment remains an exciting prospect for future management of ARHR2 and GACI secondary to loss of function of ENPP1.

## Introduction

Hypophosphatemic rickets is a heterogenous group of diseases characterized by renal phosphate wasting leading to impaired bone mineralization. Important regulators of phosphate metabolism include fibroblast growth factor 23 (FGF23), parathyroid hormone (PTH), calcitriol (1,25(OH)_2_D) and, relevant to the presented cases, ectonucleotide pyrophosphate/phosphodiesterase 1 (ENPP1).

FGF23 is a bone-derived phosphaturic hormone, mediating its effects on the metabolism of phosphate by a number of mechanisms. FGF23 increases renal phosphate excretion and suppresses synthesis of 1,25(OH)_2_D.^1^ FGF23 directly inhibits the proximal tubule renal sodium-dependent phosphate cotransporter with resultant phosphaturia. FGF23 also indirectly reduces intestinal phosphate absorption by lowering the serum level of 1,25(OH)_2_D. In particular, FGF23 reduces both the activity of renal 1-alpha-hydroxylase which synthesizes 1,25(OH)_2_D from 25-hydroxy vitamin D (25(OH)D) and increases the activity of renal 24-hydroxylase which degrades 1,25(OH)_2_D. In addition, FGF23 exerts direct actions on the parathyroid gland, inhibiting PTH gene expression and secretion.^1^ A reduction in circulating PTH concentrations further reduces the activity of 1-alpha-hydroxylase and increases the activity of 24-hydroxylase, further reducing serum 1,25(OH)_2_D.

ENPP1, a type II transmembrane glycoprotein, hydrolyses adenosine triphosphate (ATP) into adenosine monophosphate (AMP) and inorganic pyrophosphate (PPi).^2^ PPi is a potent inhibitor of calcification and tissue mineralization.^3^ PPi is degraded to inorganic phosphate (Pi) by tissue non-specific alkaline phosphatase.^4^ The balance between PPi and Pi plays a pivotal role in vascular calcification development. Reduced production of PPi may lead to pathological calcifications and aberrant tissue mineralization.

Hypophosphatemic rickets can be divided into FGF23-mediated and non-FGF23-mediated forms.^5^ The most common form of FGF23-mediated hypophosphatemic rickets is X-linked hypophosphatemia (XLH), caused by loss-of-function pathogenic variants in *PHEX.*^6^ Additional inheritable forms include autosomal dominant hypophosphatemic rickets caused by a variant in *FGF23* and three forms of autosomal recessive hypophosphatemic rickets caused by variants in *dentin matrix protein 1* (*DMP-1*) (autosomal recessive hypophosphatemic rickets type 1, ARHR1), *ENPP1* (ARHR2) or *family with sequence similarity 20, member C* (*FAM20C*) (autosomal recessive hypophosphatemic rickets type 3, ARHR3).^6^^,^^7^ Non-FGF23 mediated forms of hypophosphatemic rickets include autosomal recessive hereditary hypophosphatemic rickets with hypercalciuria secondary to biallelic variants in the *solute carrier family 34-member 3 (SLC34A3)* gene encoding the sodium-dependent phosphate transport protein 2C. In addition, X-linked recessive hypophosphatemic rickets is associated with pathogenic variants in *chloride voltage-gated channel 5* (*CLCN5*)*.* Additionally, variants in *SLC34A1* and *glucose transporter 2* (*GLUT2*) may cause FGF23-independent renal phosphate wasting.

Generalised arterial calcification of infancy (GACI) and autosomal recessive hypophosphatemic rickets type 2 (ARHR2) are caused by loss-of-function pathogenic variants in *ENPP1*. ENPP1 deficiency is known to have an estimated genetic prevalence of approximately 1:64 000 to 1:200 000.^2^ Whilst GACI and ARHR2 are recognized as two distinct clinical phenotypes which manifest at different ages, they both represent different parts of the spectrum of *ENPP1* deficiency.^8^

Herein, we present six cases of *ENPP1* loss-of-function variants causing ARHR2 and/or GACI. These cases add to the phenotypic spectrum of this exceedingly rare condition and highlight diagnostic and therapeutic challenges faced by clinicians. In addition, the unique combination of an *ENPP1* and *ALPL* loss-of-function pathogenic variant (as seen in Case 1 and 2) offers novel insights into both the pathogenesis and phenotype of this condition.

## Materials and methods

### Genetic testing

Case 1 and 6: Whole exome massively parallel sequencing, with bioinformatically targeted analysis of a hypophosphatemic rickets gene panel (genes analyzed: *ALPL, CYP27B1, CYP2R1, DMP1, ENPP1, FAM20C, FGF23, OCRL, PHEX, SLC34A1, SLC34A3, VDR*) was undertaken. Target enrichment was carried out using TWIST Exome Panel (102032) and TWIST EF Library prep kit (101 901 or 103 904). Massively parallel sequencing and primary analysis was performed on an Illumina NextSeq550 with High Output Kit v2.5 (20024908). Secondary analyses were performed using DRAGEN Enrichment v3.6.3 with GRCh38 alignment. Tertiary analyses were completed using Alissa Interpret v5.3 (Agilent Technologies) and associated annotation sources. Test sensitivity: SNP ≥ 99%, INDELS >89%; ≥95% of target sequences, including coding and ~10 bp of flanking intronic sequences were covered to a depth of at least 20x. Nomenclature is in accordance with the Human Genome Variation Society guidelines (http://varnomen.hgvs.org/). Variants are classified according to American College of Medical Genetics and Genomics (ACMG) guidelines.^9^

Case 2: Targeted genomic DNA Sanger sequencing of the *ENPP1* and *ALPL* genes was performed.

Case 3 and 5: Whole exome sequencing library preparation was performed using a TWIST BioScience Library Preparation EF KIT kit, with libraries analyzed on an Illumina NovaSeq 6000, at a multiplex of 96 exomes per S2 flow cell. Reads are aligned to Human Genome Reference Sequence GRCh38, and single nucleotide and short insertion/deletion variants identified using the Dragen Server in Illumina Basespace DRAGEN Enrichment 3.9.5. Variant filtering, prioritization and reporting are performed using the Genomics Annotation and Interpretation Application in-house pipeline. Variants are filtered based on inheritance pattern, frequency, zygosity, impact and in silico pathogenicity scores. The data analysis pipeline is based on Gemini v18 with annotation from VEP and dbNSFP. Variants are classified following the joint consensus recommendations of the American College of Medical Genetics and Genomics and the Association for Molecular Pathology for the interpretation of sequence variants (PMID: 25741868) as well as integrating guidance from the best practice guidelines for variant interpretation published by the Association for Clinical Genomic Science (https://www.acgs.uk.com/quality/best-practice-guidelines/). Variants are reported according to HGVS nomenclature.

Case 4: The Blueprint Genetics Hypophosphatemic Rickets Flex Panel (version 1, 7 January 2022) Plus Analysis including sequence analysis and copy number variation analysis of the following genes (*ALPL, CYP27B1, CYP2R1, DMP1, ENPP1, FAM20C, FGF23, PHEX, SLC34A1, SLC34A3, and VDR*) was undertaken. This panel targets protein coding exons, exon-intron boundaries and selected non-coding, deep intronic variants. Nomenclature is in accordance with the Human Genome Variation Society guidelines (http://varnomen.hgvs.org/). Variants are classified according to ACMG guidelines.^9^

### Measurements

Case 1, 2: Serum parathyroid hormone (PTH) and 25-hydroxy vitamin D (25(OH)D) were measured using an Abbott ARCHITECT i2000SR analyzer.

Case 3: Biochemistry (at the time of diagnosis) was not collected as the diagnosis was made antenatally.

Case 4: Serum PTH was measured using a Roche PTH STAT Elecsys analyzer. 25(OH)D was measured using an Alinity chemiluminescent microparticle analyzer.

Case 5: PTH was measured using a Siemens Immulite 2000Xpi platform analyser. 25(OH)D was measured using liquid chromatography tandem mass spectrometry (LC–MS/MS).

Case 6: PTH and 25(OH)D were measured using an Abbott ARCHITECT i2000SR analyzer.

## Case series

Presented below are the six case studies, further summarized in Table 1.

**Table 1:** Phenotypic and genetic details of Cases 1-6.

	Case 1	Case 2	Case 3	Case 4	Case 5	Case 6
**Age/Sex**	5-yr-old male	21-yr-old female	7-yr-old female	11-yr-old female	4-mo-old male	5-yr-old male
**Referring complaint**	Rickets (at 5yr)	Rickets (noted at 10yr, formally diagnosed at 19yr)	Antenatal diagnosis	Lower limb pain and deformity (at 7yr)	Cardiogenic shock (at 3 mo)	Shoulder discomfort since birth
**Family/social history**	Ethnicity: Afghani Parental consanguinity Older sister: short stature, leg bowing, bone/joint pain Born in Pakistan	Ethnicity: Afghani Parental consanguinity Younger brother: short stature, leg bowing, bone/joint pain Born in Afghanistan	Ethnicity: Indian No parental consanguinity Older sibling (female) deceased at 4 d from GACI complications which was diagnosed postnatally	Ethnicity: Cambodian	Ethnicity: Pakistani Parental consanguinity (first cousins) No significant family history	Ethnicity: Pakistani Parental consanguinity (first cousins) No significant family history
**Antenatal/neonatal history**	Term baby Macrocephaly, poor weight gain Development normal except walked at 2 yr	Term baby Normal development	Antenatal diagnosis Treated with etidronate from 29 wk gestation Born via LSCS at 37 wk Birthweight 2.55 kg	Term baby Normal growth and development Pain in legs	Born at 37 wk Required respiratory support at birth with Apgar scores 5,5 CPAP for 2 wk then discharged home No significant antenatal history	Term baby Pain on movement of shoulder noted on Day 1 of life Gross motor delay, speech delay
**Biochemistry at diagnosis**	Calc corr* 2.28 mmol/L (2.20-2.65) Phosphate 0.61 mmol/L (1.1-2.0) ALP 170 U/L (120-370) PTH 11 pmol/L (1.6-9.0) (Abbott Architect i2000SR) 25(OH)D 57 nmol/L (>50) (Abbott Architect i2000SR) TmP/GFR^+^ 0.583 (low)	Calc corr* 2.3 mmol/L (2.10-2.60) Phosphate 0.65 mmol/L (0.75-1.65) ALP 32 U/L (35-140) PTH 5.6 pmol/L (1.6-9.0) (Abbott Architect i2000SR) 25(OH)D 92 nmol/L (>50) (Abbott Architect i2000SR) TmP/GFR^+^ 0.760 (low)	N/A (Antenatal diagnosis)	Calc corr* 2.3 mmol/L (2.2-2.6) Phosphate 0.7 mmol/L (1.0-2.0) ALP 564 U/L (80-360) PTH 7.8 pmol/L (1.6-7.0) (Roche PTH STAT Elecsys immunoassay) 25(OH)D 26 nmol/L (50-150) (Alinity chemiluminescent microparticle immunoassay) Normal urine Ca:Cr ratio	Calc corr* 2.57 mmol/L (2.2-2.8) Phosphate 1.55 mmol/L (1.5-2.75) ALP 268 U/L (120-550) PTH 2.4 pmol/L^1–7^ (Siemens Immulite 2000Xpi platform) 25(OH)D 36 nmol/L (>50) (LCMS-MS)	Calc corr* 2.55 mmol/L (2.2-2.65) Phosphate 1.78 mmol/L (1.5-2.75) ALP 324 U/L (120-550) PTH 2.1 pmol/L (0.7-9.0) (Abbott Architect i2000SR) 25(OH)D 33 nmol/L (>50) (Abbott Architect i2000SR) Renal phosphate threshold <0.65 mmol/L (0.75-1.35)
**Rickets**	Yes – presenting complaint	Yes – presenting compliant	Yes	Yes	Yes – resolving	Yes – noted at 15 mo of age, formally diagnosed at 2 yr of age
**Arterial calcification**	Yes Aortic and pulmonary arterial calcification	Not investigated to date	Yes Extensive calcifications present at birth that improved with age: o Cardiopulmonary, thoracic, and abdominal aortic vessels Calcification of ligamentum flavum, intervertebral discs, posterior cervical spine bodies Peri-articular calcification of right shoulder limiting movement and causing radial nerve palsy Subsequent development of: o Internal carotid narrowing (particularly severe in left internal carotid artery with external carotid artery collateralization) Increased renal artery resistive indexes.	No, not documented to date	Yes Widespread calcifications of large and medium-sized arteries of the chest and abdomen including: o Descending aorta and its branches Supra-aortic branches, superior mesenteric arteries, renal arteries and external iliac arteries Coronary arteries and pulmonary arteries (partially calcified) Linear calcifications of the sternoclavicular joints	Yes – resolved Multifocal calcification of mediastinum, aorta, glenohumeral joints, sternoclavicular joints and vertebral spinous processes Improved without treatment over first year of life
**Nephrocalcinosis**	Yes	Not investigated to date	Yes	Yes	No	Yes, mild
**Dental issues?**	Yes Loss of multiple complete teeth with root intact	Yes Loss of multiple complete teeth with root intact	Yes Generalized dental hypoplasia	Yes Dental crowding, missing premolars	No	No
**Other Clinical features**	Valgus deformity of lower limbs Knee pain Abnormal gait Short stature	Episodic arthralgias predominantly affecting bilateral wrists, elbows, proximal and distal interphalangeal joints Decline in grip strength	Unilateral coronal craniosynostosis Conductive hearing loss requiring hearing aids Mild speech delay Expressive aphasia and right hemiparesis involving arm and face	Valgus deformity lower limbs Fused posterior longitudinal ligament with limited flexion/extension cervical spine Thickened left clavicle Short stature Mild conductive hearing loss	Distinctive ear structure bilaterally, no earlobe calcifications noted	Conductive hearing loss requiring hearing aids Short stature Bone pain (exacerbated by walking long distances) Abnormal gait
**Genetics**	Homozygous pathogenic *ENPP1* variant (ARHR type 2): c.783C>G p.(Tyr261*) Heterozygous pathogenic ALPL variant	Homozygous pathogenic *ENPP1* variant (ARHR type 2): c.783C>G p.(Tyr261*) Heterozygous pathogenic ALPL variant	Homozygous pathogenic *ENPP1* variant (GACI): c.749C>T p.(Pro250Leu)	Homozygous, likely pathogenic *ENPP1* variant (ARHR type 2): C.403_404del.p.(Asp135Leu)	Homozygous *ENPPI* pathogenic variant (GACI) c.876_880del.p. (Ser292Argfe*4)	Homozygous *ENPP1* pathogenic variant c.2467del.p.(Gln823Lysfs*4)
**Management**	Calcitriol and oral phosphate with normalization of phosphate levels No management required of arterial calcification or nephrocalcinosis	Calcitriol and oral phosphate	Bisphosphonates from birth until 8 mo (pamidronate then risedronate, ceased due to social reasons) Phosphate and calcitriol once diagnosed with rickets at 2yr Aspirin (commenced at 5yr due to internal carotid narrowing)	Calcitriol (60 ng/kg/d 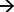 20 ng/kg/d) and phosphate (40 mg/kg/d) Potassium citrate 300 mmol TDS (nephrocalcinosis) Iron supplementation	IV Pamidronate 0.1 mg/kg/dose weekly for 4 wk Transitioned to PO Risedronate 1 mg/kg/dose weekly for 6 wk Transitioned to PO Etidronate 20 mg/kg/d Awaiting ENPP1 enzyme replacement compassionate release	Calcitriol and oral phosphate commenced at age of 2 yr Planned to enrol in ENPP1 enzyme replacement trial

*Calcium corr is corrected calcium, calculated as: total calcium (mmol/L) + [(40 − albumin (g/L)) × 0.02]; ^+^TmP/GFR calculated according to the AACB TmP/GFR Harmonization Working Group. TRP = 1 − [(serum creatinine × urine phosphate)/(serum phosphate × urine creatinine × 1000)], if TRP ≤ 0.86, TmP/GFR = TRP × serum phosphate. If TRP >0.86, TmP/GFR = {0.3 × TRP/[1 − (0.8 × TRP)]} × serum phosphate. Assumes serum creatinine value in μmol/L, serum phosphate in mmol/L, urine creatinine in mmol/L and urine phosphate in mmol/L.

Abbreviations: GACI, generalized arterial calcification of infancy; CPAP, continuous positive airway pressure; LSCS, lower uterine segment cesarean section; ALP, alkaline phosphatase; PTH, parathyroid hormone; 25(OH)D, 25-hydroxy vitamin D; Ca:Cr, calcium: creatinine ratio; TmP/GFR, tubular maximum reabsorption of phosphate to GFR; ENPP1, ectonucleotide pyrophosphate/phosphodiesterase 1; ARHR, autosomal recessive hypophosphatemic rickets.

### Case 1

A 5-yr-old male presented with a 12-mo history of worsening valgus deformity and leg pain. Skeletal survey demonstrated widespread rachitic changes at long bone metaphyses, normal skull (no Wormian bones) and normal vertebral bodies. His parents were consanguineous, and his older sister had rickets. Clinical improvement was seen with oral calcitriol and phosphate. Genetics confirmed a homozygous pathogenic variant c.783C>G p.(Tyr261*) in *ENPP1* and a heterozygous pathogenic *ALPL* variant consistent with hypophosphatasia. Both parents were confirmed as carriers of the *ENPP1* variant and the *ALPL* variant was paternally inherited. Further history revealed loss of complete teeth with intact roots. Subsequent investigations revealed aortic and pulmonary arterial calcification and bilateral medullary nephrocalcinosis, currently not requiring treatment.

### Case 2

A 21-yr-old female presented at 10 yr of age with tibial bowing and knee pain. She was reviewed by the Orthopaedic team and offered corrective surgery but declined. She subsequently developed episodic arthralgias involving the joints of her upper limbs but did not seek medical review. She was referred to adult endocrinology services at 19 yr of age, after her younger brother (Case 1) was found to have pathogenic variants in *ENPP1* and *ALPL.* Skeletal imaging of her lower limbs displayed bilateral distal femoral shaft lateral angulation and proximal tibial medial angulation, with metaphyseal transverse sclerotic bands involving the distal medial femur, proximal medial tibia and distal tibia. Clinical improvement was noted following commencement of oral calcitriol and phosphate. Familial variant testing of the *ENPP1* and *ALPL* genes confirmed the same homozygous *ENPP1* variant and heterozygous *ALPL* variant identified in her brother (Case 1). Further history revealed loss of complete teeth with intact roots. The presence of arterial calcification has not yet been investigated.

### Case 3

**Figure 1:**
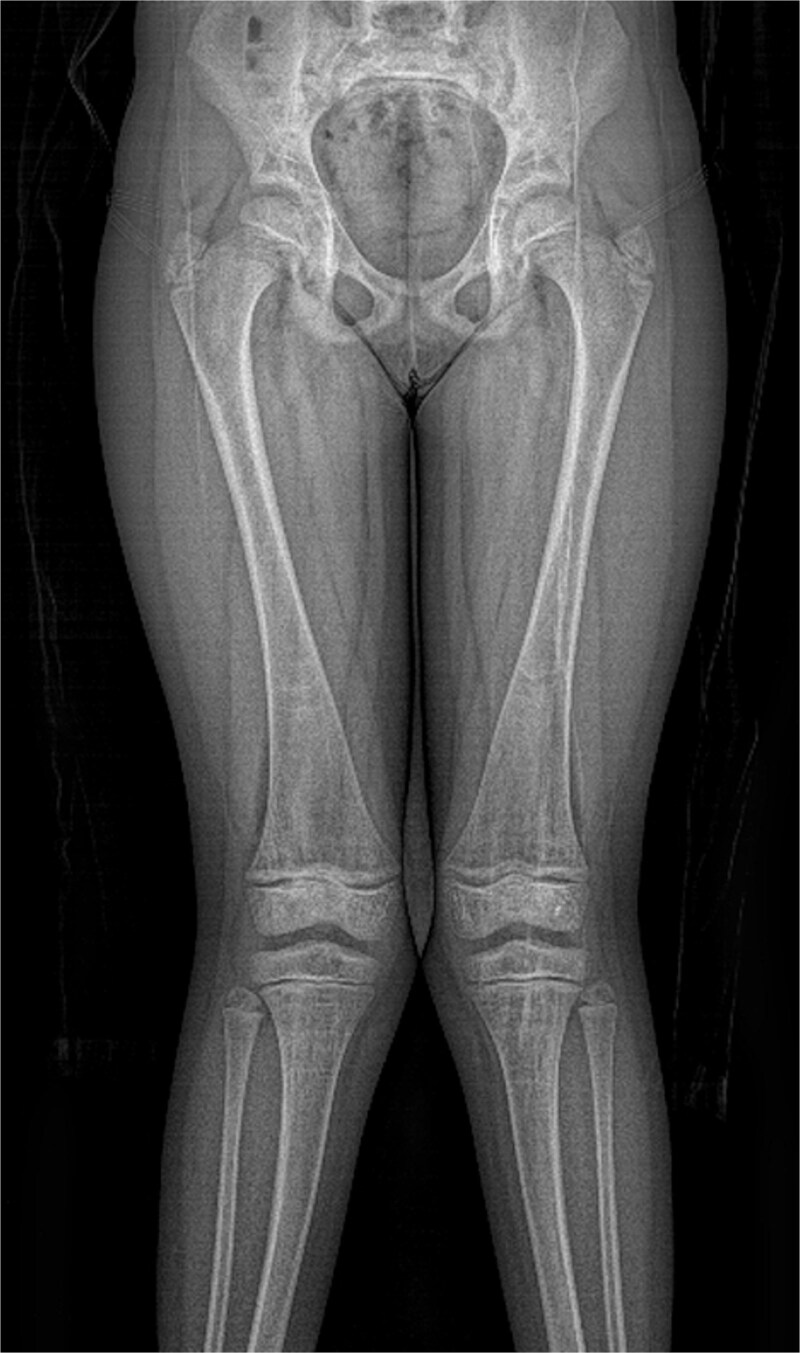
Radiograph demonstrating bowing of both femora and the tibiae. Bilateral coxa Valga.

**Figure 2:**
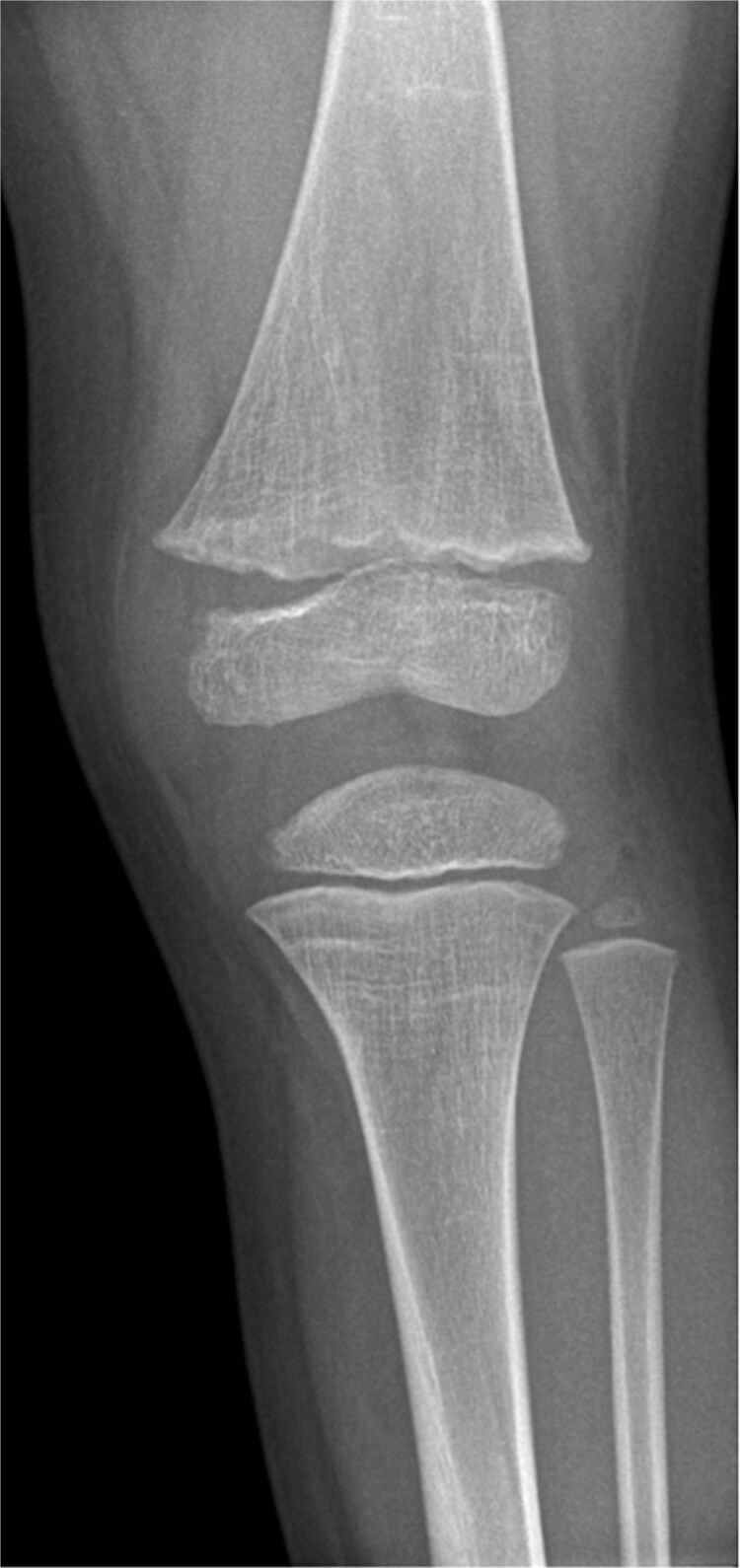
Radiograph demonstrated generalized changes of rickets with flaring and cupping of the metaphyses.

A clinical diagnosis of GACI was made antenatally. Calcifications were noted on USS imaging at 29-wk’ gestation, in the context of an older affected sibling (confirmed homozygous pathogenic variant c.749C>T p.(Pro250Leu) in *ENPP1*) who had died at 4 days of life from GACI. Maternal etidronate treatment was commenced from 29-wk’ gestation. Extensive arterial calcifications were present at birth in cardiopulmonary, thoracic and abdominal aortic vessels. Post-natal genetic testing confirmed the same homozygous *ENPP1* variant identified as in her older sibling. Bisphosphonate treatment was commenced (pamidronate, followed by risedronate) following birth and continued until 8 mo of age. Whilst the calcifications gradually improved, she subsequently developed rickets (Figures 1 and 2). Phosphate and calcitriol were commenced at 2 yr of age. Ongoing issues include nephrocalcinosis (likely due to calcitriol treatment), internal carotid narrowing requiring aspirin commencement at five years of age and increased renal artery resistive indices. In addition, generalized dental hypoplasia was evident, requiring crown placement. More recently, she presented at the age of 7 yr with focal status epilepticus, aphasia and right sided hemiparesis. Magnetic resonance imaging (MRI) and cerebral angiography demonstrated ischemia in the left parietal lobe with narrowing of the left internal carotid artery and basilar artery. Neurological improvement was observed following indirect encephaloduroarteriomyosynangiosis revascularization.

### Case 4

A 7-year-old female presented with knee pain, fixed bilateral flexion knee deformities, limited cervical spine movement, and thickened left clavicle. A homozygous, likely pathogenic, variant c.403_404del.p.(Asp135Leu) in *ENPP1* was confirmed. She was managed with calcitriol and phosphate. She developed nephrocalcinosis requiring potassium citrate therapy. Arterial calcification has not been identified, and blood pressure remains normal.

### Case 5

A 3-month-old male presented to the general practitioner with concerns of poor feeding and abnormal movements. He was referred to the Emergency Department where he had rapid respiratory deterioration and required cardiac compressions. On transfer to a tertiary centre neonatal intensive care unit (NICU) he developed cardiogenic shock with absent pulses. Computed tomography (CT) imaging of his abdomen and pelvis displayed widespread calcifications of large and medium-sized arteries. A homozygous *ENPP1* pathogenic variant c.876_880del.p. (Ser292Argfe*4) was confirmed. He was commenced on bisphosphonate treatment (pamidronate, risedronate) prior to genetic confirmation and continues on 1st generation bisphosphonate therapy (etidronate). Compassionate access to ENPP1 enzyme replacement is in progress.

### Cass 6

**Figure 3:**
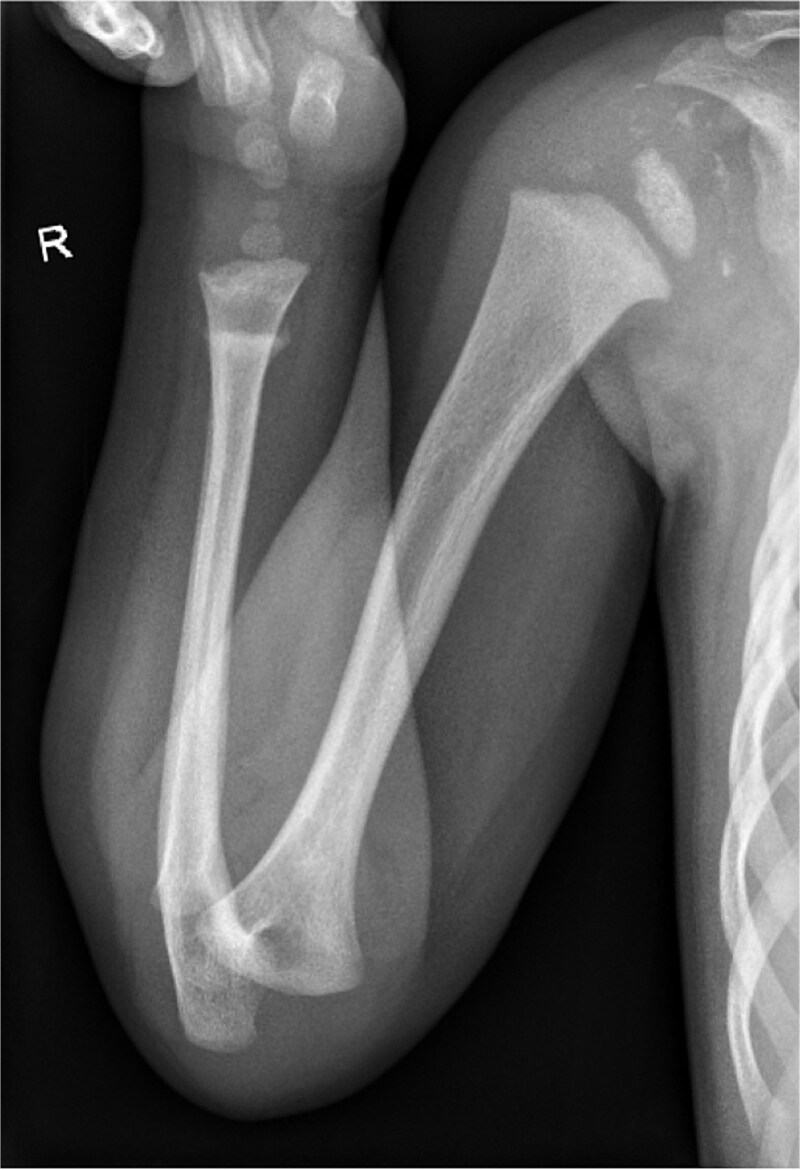
Radiographs demonstrating extensive right glenohumeral peri-articular calcification.

**Figure 4:**
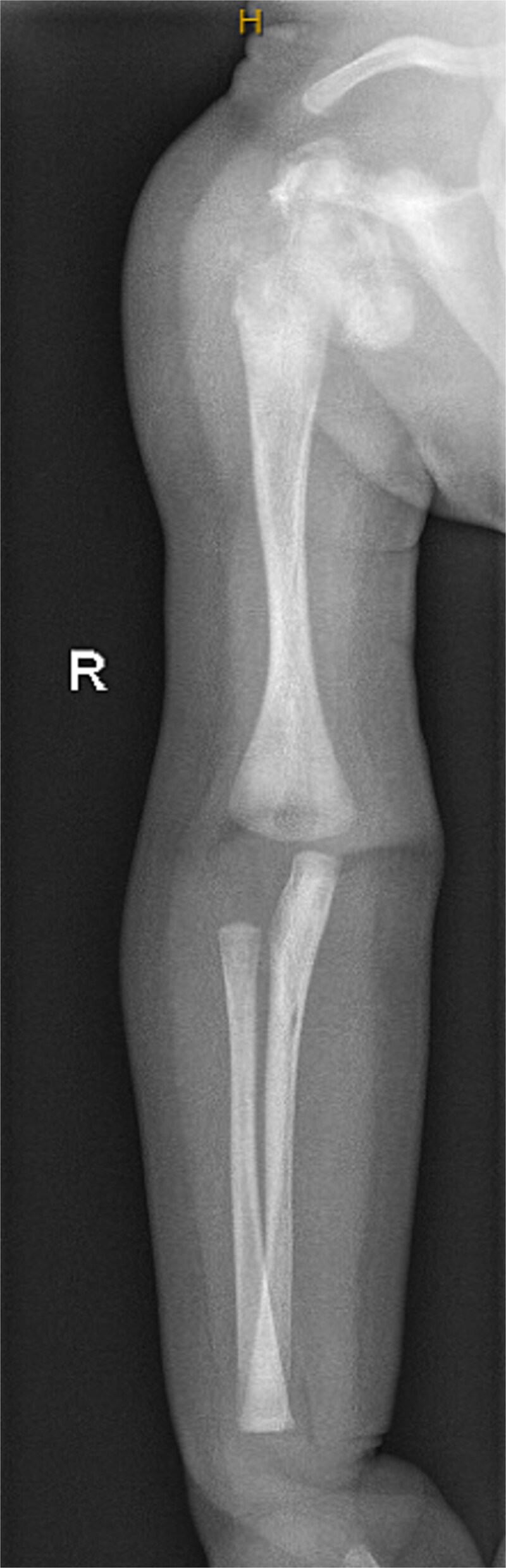
Radiographs demonstrating extensive right glenohumeral peri-articular calcification.

**Figure 5:**
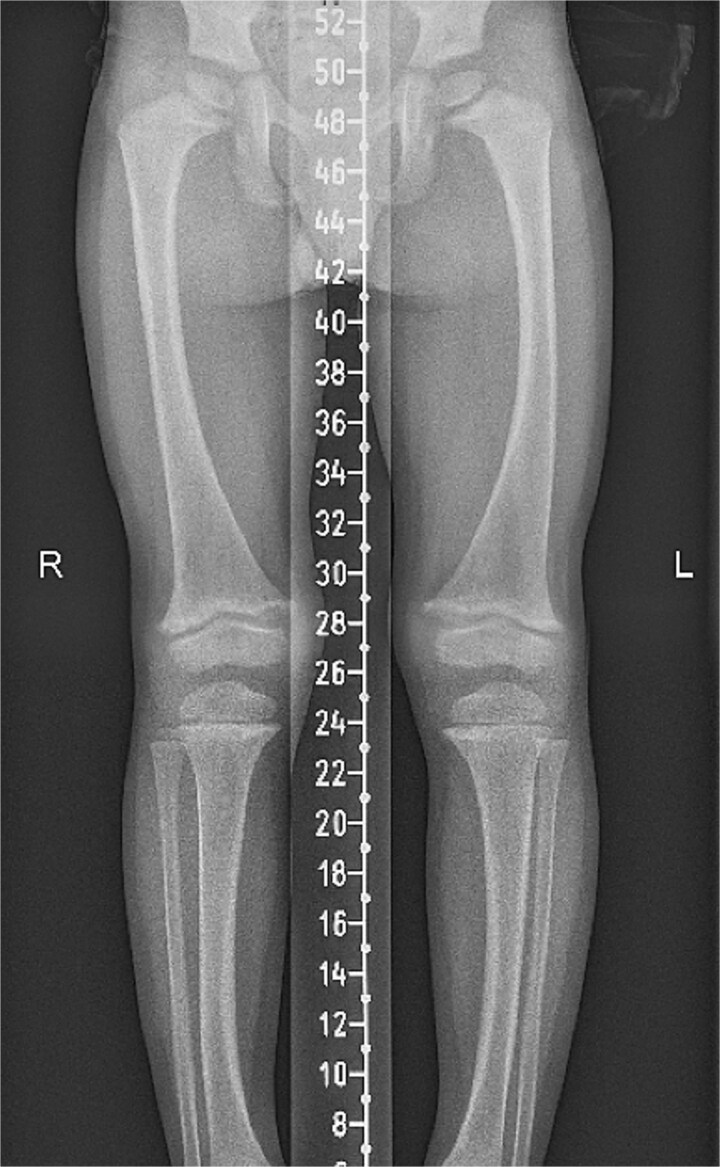
Radiograph demonstrating mild bowing of both femora and tibiae, noting underlying generalized changes of rickets with flaring and cupping of the metaphyses. Borderline bilateral coxa vara.

A 2-wk-old infant initially presented with right shoulder discomfort noted since birth. An X-ray revealed deformity and calcification of the right shoulder joint (Figures 3 and 4). MRI demonstrated extensive pericapsular calcification of the glenohumeral joint. Further CT imaging revealed extensive multifocal vascular calcification of the aorta, mediastinum, sternoclavicular joint and cervical and thoracic spinous processes. There was no evidence of cardiovascular compromise. Echocardiogram and CT cardiac angiogram did not display coronary artery calcification. Renal ultrasound displayed dysplastic kidneys. A homozygous *ENPP1* pathogenic variant c.2467del.p.(Gln823Lysfs*4) was confirmed. Calcitriol and oral phosphate were commenced at 2 yr of age following the emergence of hypophosphatemia, renal phosphate wasting and clinical and radiographic evidence of rickets (Figure 5). Enrolment in an ENPP1 enzyme replacement trial is planned.

## Discussion

The 6 cases presented highlight the diverse phenotype, diagnostic process and clinical management of ENPP1 deficiency. Cases 1, 2, and 4 presented at the age of 5-, 10-, and 7-yr, respectively, with lower limb deformities consistent with rickets. Case 3 was diagnosed antenatally following an older sibling dying from complications of GACI at four days of life. Case 5 presented acutely unwell at the age of 3-months in cardiogenic shock with marked arterial calcifications. Case 6 presented at the age of 2-wk with shoulder discomfort since birth. Whilst all cases have evidence of hypophosphatemic rickets, the burden of arterial calcification is markedly different between the cases (Case 1; aortic and pulmonary arterial calcification, Case 2; yet to be investigated, Case 3; extensive cardiopulmonary, ligamentous, thoracic, abdominal calcification, Case 4; nil calcification, Case 5; widespread thoracic and abdominal arterial calcification and Case 6; resolved mediastinal, aortic, glenohumeral and vertebral calcification). Cases 1, 2, 3 and 4 had dental concerns. Whilst Cases 1, 2, 3, 4 and 6 required calcitriol and phosphate treatment, bisphosphonate treatment was only instituted in the management of Cases 3 and 5. ENPP1 enzyme replacement therapy has been proposed in Cases 5 and 6.

Biallelic loss-of-function pathogenic variants in *ENPP1* can result in two key clinical phenotypes; GACI and ARHR2.^6^^,^^7^^,^^10^ GACI is a life-threatening condition, often presenting in the neonatal period with heart failure and arterial hypertension, typified by calcification of the media in large- and medium-sized arteries and intimal proliferation.^6^^,^^11^ Arterial stenoses may ensue, leading to compromised blood flow and myocardial or cerebral infarctions.^11^ In addition to variants in *ENPP1*, pathogenic variants in *ABCC6* have been associated with GACI.^12^

The phosphate wasting of ARHR2 develops based on the same genetic alterations in *ENPP1* as GACI, but typically manifests later in life. Significant clinical variability, as demonstrated in the presented cases, is seen in both conditions, as well as a lack of genotype–phenotype correlation.^7^^,^^11^ Several reports have even described divergent clinical courses in siblings with identical homozygous *ENPP1* pathogenic variants.^13^^,^^14^ Children with ARHR2 commonly exhibit short stature, early fusion of cranial sutures, rachitic skeletal changes, progressive varus deformity of the lower limbs, skeletal fragility and bone and muscle pain. Furthermore, dental abnormalities (eg, abscess and thin enamel and dentin) are commonly seen in patients with hypophosphatemic rickets.^15^ In addition to ARHR2 and GACI, loss-of-function pathogenic variants in *ENPP1* can lead to progressive mixed hearing loss, ossification of the posterior longitudinal ligament, pseudoxanthoma elasticum (PXE), thrombocytopaenia, hypoglycaemia, neurologic and hepatic manifestations.^6^^,^^16–18^ Furthermore, monoallelic *ENPP1* variants have been implicated in case reports of adults with early onset osteoporosis and fractures.^19–21^

*ENPP1* plays a critical role in vascular calcification and FGF23 activity. Loss-of-function *ENPP1* variants lead to reduced production of PPi, a key inhibitor of hydroxyapatite crystal deposition.^2^^,^^4^^,^^17^ Murine models have demonstrated, following the administration of ENPP1-Fc, elevated plasma PPi levels from nearly undetectable to those seen in wildtype sibling pairs.^22^ The reduced production of PPi may predispose to precipitation of calcium and phosphorus to form calcium phosphate with resultant pathological calcifications and unregulated tissue mineralization.^22^ In addition, the vascular stenosis observed in GACI is not solely due to calcium deposition, but also vascular smooth muscle cell proliferation, further reducing lumen diameter and compromising blood flow.^23^ Lack of AMP generation in ENPP1 deficiency has been postulated to cause the neointimal proliferation.^23^^,^^24^ ENPP1-Fc fusion protein administration to mice models of GACI has been shown to eliminate cardiac and arterial calcifications, reduce hypertension, prevent the formation of neointimal proliferation and improve survival.^22^^,^^24^^,^^25^

Bone microarchitecture, mineralization and FGF23 expression is altered by ENPP1 deficiency. *ENPP1*^-/-^ knockout mice display reduced cortical and trabecular bone mass, reduced osteocyte lacunar size, narrowed canaliculi and decreased mechanical loading^23^^,^^26^^,^^27^ compared to wild type. In addition, similar to humans, *ENPP1*^-/-^ knockout mice display greater expression of FGF23 mRNA compared to wild type mice.^26^ The underlying mechanism linking ENPP1 and FGF23 is not well understood, however it has been postulated and demonstrated via murine models that *ENPP1* suppresses *FGF23* gene expression.^26^^,^^28^^,^^29^

Case 1 and 2 (siblings) demonstrated the novel combination of simultaneous loss-of-function pathogenic variants in *ENPP1* and *ALPL*. As detailed above, variants in *ENPP1* lead to reduced production of PPi, a potent inhibitor of calcification and mineralization. PPi is degraded by tissue non-specific alkaline phosphatase (TNSALP) to inorganic phosphate.^4^ Hypophosphatasia is caused by mono- or biallelic pathogenic variants in the *ALPL* gene encoding the TNSALP protein.^30^ We postulate that the severity of Case 1 and 2’s *ENPP1* phenotype may be ameliorated by the concurrent *ALPL* pathogenic variant due to the theoretically higher levels of PPi in the setting of impaired TNSALP activity. The dental issue described in Cases 1 and 2, loss of complete teeth with intact roots is consistent with various forms of hypophosphatasia (HPP), including childhood HPP, adult HPP and odontohypophosphatasia, an isolated dental form.^3^^,^^31^ It is not clear whether this *ALPL* loss-of-function variant is in keeping with HPP or odontohypophosphatasia, or the degree to which it may ameliorate the ENPP1 phenotype. In addition, diagnosis of hypophosphatasia is typically suspected by low serum alkaline phosphatase levels. However, the accurate interpretation of serum alkaline phosphatase levels can be misleading when FGF23 is elevated (FGF23 suppresses alkaline phosphatase). Genetic analyses are critical to exclude a diagnosis of hypophosphatasia.

The diagnoses of ARHR2 and GACI are typically made based on a combination of clinical features, biochemical results, imaging and genetic analyses.^6^ ARHR2 manifests during childhood with bone pain, myalgia, short stature, rachitic skeletal deformities and lower limb abnormalities.^6^ Serum concentrations of 1,25(OH)_2_D are inappropriately low or normal in the setting of hypophosphatemia. 25(OH)D and calcium are typically normal, PTH normal or mildly elevated (as demonstrated in Cases 1 and 4), serum alkaline phosphatase elevated, whilst tubular reabsorption of phosphate is diminished.^5^^,^^15^^,^^32^ Renal phosphate wasting should be evaluated by calculating the tubular maximum reabsorption of phosphate per glomerular filtration rate (TmP/GFR).^33^ Serum intact FGF23 levels are likely elevated or inappropriately normal in the setting of hypophosphatemia.^34^ Radiographs can also provide important diagnostic clues. Ultimately, genetic analysis is required to confirm the diagnosis due to the significant clinical heterogeneity of ENPP1 deficiency.^19^ In addition, genetic analysis may reveal, or exclude, additional concurrent pathogenic variants (as demonstrated in Cases 1 and 2).

Conventional treatment (as in Cases 1-4 and 6) of ARHR2 predominantly centres upon oral phosphate and active vitamin D.^7^ Boyce et al., proposed initial doses of calcitriol at 15 ng/kg/d and phosphate at 25–30 mg/kg/d.^23^ Drusun et al., recently described initial doses of calcitriol at 15–50 ng/kg/d and phosphate at 20–145 mg/kg/d.^7^ Treatment aims include correction of clinical findings and normalization of serum alkaline phosphatase (rather than normalization of serum phosphate). Of note, serum alkaline phosphatase levels can be affected by concomitant pathogenic variants in the *ALPL*-gene. Regular monitoring for recognized side effects of treatment including nephrocalcinosis and hyperparathyroidism^7^^,^^23^^,^^32^ should be undertaken. Previously, concern was raised regarding phosphate replacement due to the association between hypophosphatemia and hyperphosphaturia with increased survival in GACI patients.^35^^,^^36^ Multiple subsequent reports have described stable long-term follow-up of GACI patients without worsening of calcification following phosphate supplementation.^11^^,^^35^

Bisphosphonates have been utilized in the treatment of GACI for over 40 yr. First-generation bisphosphonates (eg, etidronate), in comparison to newer bisphosphonates with greater antiresorptive potency (eg, pamidronate, alendronate, zoledronate) have been postulated to be superior in the management of GACI due to the therapeutic goal of reduced bone mineralization (rather than bone resorption).^23^ Whilst bisphosphonates, synthetic PPi analogs, certainly make physiological sense, their value remains controversial.^8^^,^^37^ Indeed, although mice models have demonstrated improvement in ectopic mineralization of skin and aorta following bisphosphonate administration, their clinical benefit is less clear.^38^ Rutsch et al.*,* reported a survival rate of 65% in bisphosphonate-treated (etidronate/pamidronate/clodronate/risedronate) GACI patients versus 31% in GACI patients not treated with bisphosphonates.^36^ The validity of this data has been questioned as the most severely affected patients who had already died were not included.^23^ In addition, it is unclear whether resolution of the vascular calcifications of GACI is due to the natural disease course or the benefit of bisphosphonate therapy.^37^ Spontaneous improvement of calcifications in GACI, as seen in Case 6, has been extensively described.^7^^,^^37^^,^^39^^,^^40^ Recently, Ferreira et al.*,* described no significant benefit in using bisphosphonates (etidronate/pamidronate/risedronate) in a large retrospective study of 247 patients with GACI.^8^ In addition, there is very limited data describing the antenatal use of bisphosphonates.^41^^,^^42^ Finally, the use of bisphosphonates in patients with ARHR2 has not been studied.^6^^,^^17^

Burosumab, a fully humanized immunoglobin G1 monoclonal antibody to FGF23, is predominantly used in the management of paediatric and adult patients with XLH and ameliorates many of the clinical features, including hypophosphataemia and rickets.^15^ The use of burosumab in the management of other forms of hypophosphatemic rickets has been proposed. Bai et al., reported on the use of burosumab in two adult brothers with ARHR1.^32^ The siblings experienced improved bone pain, healing of pseudo fractures and normalization of serum phosphate levels. More recently, Parolin et al.,^43^ demonstrated efficacious and safe use of burosumab (over 3 yr) in a 4-year-old female with ARHR2 without signs or history of GACI. However, the use of burosumab is highly controversial. FGF23 elevation with ensuing hypophosphatemia has been posited to possibly ameliorate arterial calcification in patients with GACI. Importantly, as FGF23 suppresses alkaline phosphatase, FGF23 inhibition may cause elevated alkaline phosphatase levels and consequent further reduction in PPi.^11^^,^^44^ Thus, there is a possibility that burosumab-induced suppression of FGF23 could potentially exacerbate calcifications.^23^ This is supported by a case report of a 15-yr-old male with GACI since birth who, following 20 months of burosumab for hypophosphatemia and genu varum deformity, demonstrated significant cardiac calcifications necessitating burosumab cessation.^45^ Overall, the use of burosumab in ARHR2 is experimental, and further dedicated studies are required. To this end, Ziegler and Ferreira have suggested that every patient with hypophosphatemic rickets being considered for burosumab treatment should undergo genetic testing to exclude ENPP1 deficiency.^46^

Lastly, ENPP1 replacement therapy holds promise as a future treatment option for ARHR2 and GACI secondary to *ENPP1* loss-of-function pathogenic variant. Cheng et al.*,* demonstrated increased PPi levels, decreased mortality and calcification in *ENPP1*-deficient mice following administration of INZ-701, a recombinant protein containing the extracellular domains of human ENPP1 fused to the Fc fragment of human IgG1.^47^ Ferreira et al.*,* reported improved bone strength and stiffness, trabecular bone mineral density and normalized osteoid volumes in *ENPP1*^-/-^ mice subsequent to daily injections of ENPP1-Fc.^10^ Phase 1/2 dose escalation studies of INZ-701 are underway in adults, children and infants with ENPP1 deficiency.^48^^,^^49^ Phase 3 studies are planned to assess the efficacy and safety of INZ-701 in children and infants with ENPP1 deficiency.^50^

In conclusion, ARHR2 and GACI represent part of the spectrum of ENPP1 deficiency. There is significant phenotypic heterogeneity of both conditions, as demonstrated by the six cases presented in this case series. Genetic testing is of importance as it ensures, (1) an accurate diagnosis of ARHR2, (2) exclusion of ENPP1 deficiency in patients with hypophosphatemic rickets being considered for burosumab therapy, and (3) diagnosis/exclusion of additional genetic conditions (ie, hypophosphatasia). Whilst conventional therapy, calcitriol and phosphate for ARHR2 and bisphosphonates for GACI, has been the mainstay for years, current work is underway to evaluate more targeted therapy.

## Data Availability

The authors confirm that the data supporting the findings of this study are available within the article. Any additional reasonable request can be made to LC, Lucy.collins@monash.edu.
